# Role of Structure and Composition on the Performances of P-Type Tin Oxide Thin-Film Transistors Processed at Low-Temperatures

**DOI:** 10.3390/nano9030320

**Published:** 2019-03-01

**Authors:** Raquel Barros, Kachirayil J. Saji, João C. Waerenborgh, Pedro Barquinha, Luís Pereira, Emanuel Carlos, Rodrigo Martins, Elvira Fortunato

**Affiliations:** 1 CENIMAT/I3N, Departamento de Ciência dos Materiais, Faculdade de Ciências e Tecnologia, FCT, Universidade Nova de Lisboa and CEMOP-UNINOVA, Campus da Caparica, 2829-516 Caparica, Portugal; rbarros@hovione.com (R.B.); saji@cusat.ac.in (K.J.S.); pmcb@fct.unl.pt (P.B.); lmnp@fct.unl.pt (L.P.); e.carlos@campus.fct.unl.pt (E.C.); 2 Hovione, Campus do Lumiar, Edifício S, Estrada do Paço do Lumiar, 1649-038 Lisboa, Portugal; 3 International School of Photonics, Cochin University of Science and Technology, Kochi – 682 022, India; 4 C2TN, DECN, Instituto Superior Técnico, Universidade de Lisboa, 2695-066 Bobadela LRS, Portugal; jcarlos@ctn.tecnico.ulisboa.pt

**Keywords:** p-type TFT, p-type oxide semiconductors, SnO electrical properties, oxide structure analysis

## Abstract

This work reports on the role of structure and composition on the determination of the performances of p-type SnO_x_ TFTs with a bottom gate configuration deposited by rf magnetron sputtering at room temperature, followed by a post-annealed step up to 200 °C at different oxygen partial pressures (O_pp_) between 0% and 20% but where the p-type conduction was only observed between in a narrow window, from 2.8% to 3.8%. The role of structure and composition were evaluated by XRD and Mössbauer spectroscopic studies that allows to identify the best phases/compositions and thicknesses (around 12 nm) to be used to produce p-type TFTs with saturation mobility of 4.6 cm^2^ V^−1^ s^−1^ and on-off ratio above 7 × 10^4^, operating at the enhancement mode with a saturation voltage of −10 V. Moreover, a brief overview is also presented concerning the present state of the existing developments in processing SnO_x_ TFTs with different methods and using different device configurations.

## 1. Introduction

Oxide electronics are a promising alternative to amorphous silicon (a-Si:H) and organic semiconductors to build reliable Thin Film Transistors (TFT) and more complex electronic circuits, addressing the challenges of flexible electronics and of the low cost and disposable electronics. In spite of the earlier work made during the 60s concerning the processing of n-type TFT [1,2], only forty five years later, with the work of Hosono [3], Wager [4], Carcia [5] and Fortunato [6], a significant worldwide interest materialised, especially for the active matrix for organic light emitting diodes (AMOLED) technology, exploiting their electronic properties, such as high saturation mobility, excellent uniformity and homogeneity, together with a high reliability associated with a low or room processing temperature [7]. 

However, there is no report on p-type oxide TFTs that are processed and cured at low temperatures with a performance similar to n- type, due to the low hole mobilities so far achieved in running stable and reliable devices [8]. The achievement of reliable p-type TFT, with performances similar to n-type TFT is of great importance for shaping electronics challenges towards the production of complementary metal oxide semiconductors (CMOS), a key device for analogic and digital electronic systems, thanks to their low power consumption. This is a noticeable relevant CMOS property for low cost flexible electronics. To this end, we could use organic semiconductors, aiming to exploit the advantage that they can be processed at low temperatures. 

Concerning organic p-type TFTs, most device performances on stability and mobility (<2 cm^2^ V^−1^ s^−1^) are low [9,10,11,12], while the n-type organic TFT still exhibits low mobilities (≤1 cm^2^ V^−1^ s^−1^) and requires a high absolute on voltages to switch it on [13,14].

An alternative to this is the inorganic oxide TFT, which is robust but in most cases requires high process temperatures. So far, most of the reported oxide TFTs are n-type, processed either on rigid or flexible substrates in which exists a consolidate set of results for films processed via physical or chemical methods [1,2,3,4,5,6,7,8,15,16]. For p-type, the transport due to holes is associated wirh oxygen *p* asymmetric orbitals, which severely limit the carrier mobility and therefore the TFT performances. In spite of Cu_2_O being a p-type oxide with mobility >100 cm^2^ V^−1^ s^−1^ [17,18], the TFT based on these thin films or their compositions as Cu:NiO, exhibit mobilities and On-Off current ratios of <1.5 cm^2^ V^−1^ s^−1^ and 10^4^ respectively [17,18,19,20,21]. Other materials have been also reported, such as NiO_x_ processed/annealed at 300 °C, exhibiting mobilities above 25 cm^2^ V^−1^ s^−1^ [22]. 

Tin oxide has been studied as an alternative material to produce p-type oxides, with similar performances as those obtained in n-type oxides. The structure, morphology and ambipolar characteristic of these films are well known for oxides processed by reactive sputtering using metal targets and heat treated at 400 °C [23]. Indeed, it is known that SnO has an indirect band gap structure specifically controlled by the divalent tin (SnII), in a layered crystal structure [24,25] with major contributions from Sn 5*s* and O 2*p* orbitals near the valence band maximum (VBM) and Sn 5*p* orbitals towards the conduction band minimum (CBM). The p-type behaviour is mainly attributed to the Sn vacancy and the O interstitial where tin is in Sn^2+^ oxidation state [24,25]. The excess oxygen in the film transforms some cations in Sn^3+^ to maintain electrical neutrality. This process is considered to be Sn^2+^ capturing a hole and forming weak bonded holes, located inside the bandgap, near the top of the valence band as localized acceptor states [26,27]. This means that the final free carriers’ behaviour of the films process is highly dependent on how oxygen is bonded and how it may compensate for defects.

Here, the contributions from Sn 5*s* states to VBM offer appreciable hole mobility in this material, without using a high processes temperature [28,29]. This leads also to the production of TFT with different geometry configurations [30] or using, besides metallic targets, ceramic ones on films grown by rf magnetron sputtering, heat treated at 400 °C [31].

In Table 1 we present the set of developments obtained concerning the performances of p-type SnO TFTs produced by Radio Frequency Magnetron Sputtering (RFMS) in the last 10 years [28,32,33,34,35,36,37,38,39,40,41,42,43,44,45,46,47,48,49]. There, we also present the architecture selected (SBG: staggered bottom-gate; STG: staggered top-gate; CBG: coplanar bottom-gate; CTG: coplanar top-gate; DG: double-gate), the process temperature, the oxygen partial pressure (O_pp_) and the type of dielectric used. 

Overall, we notice that the only devices processed at room temperature using the RFMS technique are those developed by the present group [28,33]. Here, it is also relevant to mention that the presence of low oxygen partial pressure during the deposition process enables the production of more stable devices [35,48,49]. Apart from that, the configuration most used is the staggered bottom-gate, while the most common dielectric used is the silicon dioxide. Apart from that, most of the substrates used are rigid (glass or silicon wafer), except that referred to as CMOS devices integrating p-type TFT based on SnO_x_ made on paper [33].

Besides stability and reproducibility issues, the data presented show that the device with the best mobility (5.53 cm^2^ V^−1^ s^−1^, with Perovskite-Mediated Photogating [47]) does not correspond to the device with the highest On/Off (I_on_/I_off_) ratio (5.2 × 10^6^, using argon-plasma surface treatment [45]). Apart from that, most of the TFT studied does not work on the enhancement mode, as desired for application purposes. 

Moreover, we noticed that the thickness of the channel layer, together with the state of the surface (degree of roughness and surface defects), determine the electrical characteristics presented by TFT and its stability.

In Table 2 we present the most significant data achieved in the last ten years concerning the production of SnO_x_ p-type TFT using different processing techniques such as: Pulsed Laser Deposition (PLD); Electron-Beam Evaporation (EBE); Thermal Evaporation (TE); direct current magnetron sputtering (DCMS); PVD: Physical Vapor Deposition (PVD); Atomic Layer Deposition (ALD); Spin-Coating (SC). As in Table 1, the different type of device configurations are also shown (SBG: staggered bottom-gate; STG: staggered top-gate; CBG: coplanar bottom-gate; CTG: coplanar top-gate; DG: double-gate); Oxygen partial pressures (O_pp_); dielectrics and process temperatures used.

Overall, the best p-type TFTs fabricated so far have been those processed by DCMS, exhibiting a mobility of 6.54 cm^2^ V^−1^ s^−1^ and an On/Off ratio of 10^5^, working in the depletion mode [30]. Moreover, the p-type TFT processed by PVD and using a STG configuration exhibit the highest recorded On/Off ratio (9.6 × 10^6^) [56].

From the present state of the art, we saw that there are several parameters that impact on the electrical performance presented by p-type TFT SnO_x_ based, most of them connected to the process parameters used, the structure of the films obtained, as well as the dielectric and the geometry configuration used.

In this paper, we report the fabrication of p-type SnO_x_ TFTs deposited by RFMS technique at RT that are post-annealed up to 200 °C, turning the process compatible with the use of low-cost flexible substrates as paper [7]. In this study, we aim to better understand the role that the structure, surface finishing and oxygen play during the growing process of SnO_x_ in order to define a process window that allows the production of reliable and high stable p-type TFT with high electronic performances, such as field effect mobility and On-Off- current ratios. 

## 2. Materials, Methods and Results

### 2.1. Experimental Details

#### Films Preparation

SnO_x_ thin films (5–100 nm) were deposited on glass substrates with an r.f. magnetron sputtering system at room temperature, using a metallic tin target (99.999% pure). Depositions were carried out in a controlled atmosphere of oxygen and argon, using an r.f. power of 40 W and 4 substrates of 1 inch ×1 inch placed in the substrate holder were rotated at a speed of 40 rpm, aiming to get high uniform films overall substrate area. Experiments were performed by varying O_pp_ (O_pp_ = P_O2_/(P_O2_ + P_Ar_), between 0% and 20%, where P_O2_ and P_Ar_ are partial pressures of oxygen and argon, respectively, keeping the total deposition pressure constant at 0.2 Pa. Moreover, the argon gas flow was kept constant—around 50sccm—while the oxygen gas flow varied from 0 to 12.5 sccm. At these conditions, the deposition rate was 40 Å/min. After deposition, the films were annealed under standard environment conditions at temperatures around 200 °C, for different times inside a tubular furnace.

### 2.2. Structure Morphology, Composition and Electro-Optical Data and Analysis

Prior to processing the TFT devices, the material in which the channel is based was deposited on glass substrates and their structure, morphology, composition and electro-optical properties were fully analysed, aiming to determine the best conditions in which to grow the channel layers of the TFT. The structure of the films was studied by X-ray diffraction (XRD) using a PANalytical X’Pert PRO (Cambridge, MA, USA) with Cu Kα radiation (λ = 1.540598 Å) while the morphology was assessed by scanning electron microscopy (SEM) with a ZEISS SEM/FIB AURIGA (Jena, Germany) operated at 2 kV, with an aperture size of 30 µm and a working distance of 5.2 nm. The surface roughness of the films was analysed using atomic force microscopy (AFM) with an Asylum MFP-3D instrument (Oxford Instruments, Oxford, UK) in non-contact mode.

The optical transmittance (T%) was measured between 300 to 2500 nm, using a double-beam UV–vis-NIR spectrometer (Lambda 950, San Dimas, CA, USA). 

The electrical resistivity (ρ), Hall mobility (µ) and free carrier concentration and their nature (electrons or holes) were determined by Hall effect measurements in Van der Pauw geometry in a Biorad HL 5500 equipment (York, England) using a constant magnetic field of 0.5 T. The electrical properties of the samples were measured at room temperature.

### 2.3. Structure Data and Analysis

**Figure 1:**
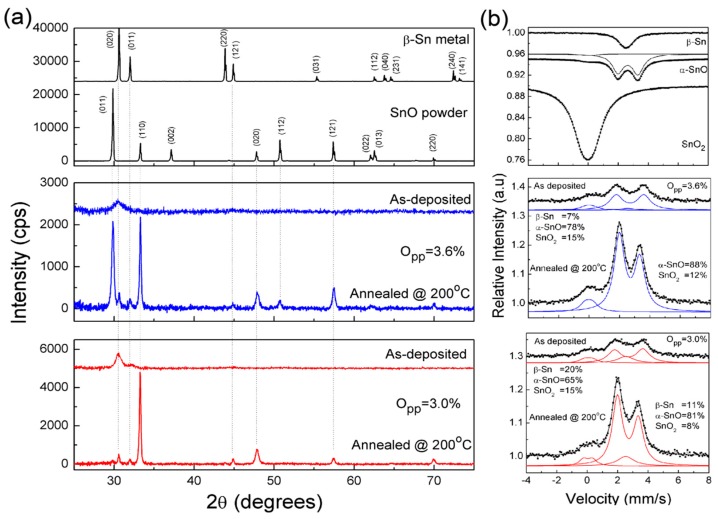
(**a**) Diffractograms of SnO_x_ thin films as-deposited. (**b**) Transmission ^119^Sn Mössbauer and CEMS spectra of as-deposited and annealed SnO_x_ films with O_pp_ = 3.0% and O_pp_ = 3.6% O_pp_, respectively.

Figure 1 shows the XRD and Mössbauer and CEMS spectra of as-deposited and annealed SnO_x_ films with O_pp_ = 3.0% and O_pp_ = 3.6% O_pp_, respectively, as the limits of the interval where a p-type transport behaviour is observed, as proven by Hall effect measurements (a positive Hall coefficient obtained for all samples evaluated, after annealing). 

The XRD data show that the films as deposited are amorphous, turning crystalline after annealing at 200 °C.

Figure 1a shows that the XRD pattern of films are 120 nm thick as deposited, where we can see that the metallic tin dominates over the SnO phase. It also shows the XRD diffractograms for SnO powder and metallic Sn, to be taken as references. 

Room temperature transmission Mössbauer and ^119^Sn conversion-electron Mössbauer spectroscopy (CEMS) were performed on the set of samples prepared, before and after annealing, using a proportional backscatter detector RIKON-5 (Wissel) in flowing 5% CH_4_-95% He gas mixture (see Figure 1b). The spectra were collected using a conventional constant acceleration spectrometer and a 5 mCi Ca^119m^SnO_3_ source. The velocity scale was calibrated using a ^57^Co (Rh) source and an α-Fe foil. The Sn isomer shifts (IS) are given relative to BaSnO_3_ reference material at 295 K (RT) and obtained by adding 0.031 mm/s to the IS relative to the source. The spectra were fitted to Lorentzian lines using a non-linear least-squares method. The set of extrapolated parameters extracted are presented in Table 3.

The Mössbauer spectra of Sn, SnO and SnO_2_ samples were also taken as reference samples in order to compare with those of the phases detected in the films by CEMS. The spectra of the reference samples reveal the typical spectra corresponding to β-Sn, α-SnO and SnO_2_, respectively [59,60]. The α-SnO spectrum reveals the typical air contamination due to the higher recoilless fraction of Sn^4+^ in SnO_2_ as compared to Sn^2+^ in α-SnO [61,62], besides the presence of the α-SnO [60,61,62,63]. The spectra recorded were fitted by three contributions connected to isomer shifts (IS) of Sn^4+^, Sn^2+^ and metallic Sn (see Table 1). The absorption peak due to metallic Sn is similar to that of the β-Sn, confirming its presence, which agreed with the XRD data. Moreover, the IS quadrupole splitting (QS) and the line widths of Sn^2+^ in the films before annealing are higher than the corresponding parameters for bulk α-SnO. After annealing, the widths of Sn^2+^ decrease, reaching values close to those of crystalline α-SnO. This suggests that the Sn^2+^ oxide present is amorphous before annealing, transforming into the crystalline form after annealing at 200 °C for at least 30 minutes. These data agree with those obtained from XRD for the same samples, showing that the films as-deposited are mainly composed of amorphous SnO and metallic β-Sn, with residual amounts of SnO_2_, which were only detected by CEMS. The SnO_2_ IS and quadrupole splitting (QS) deduced differs from those of bulk SnO_2_ which we attribute to the low degree of crystallinity of the films. Assuming that the recoilless factors of β-Sn, SnO and SnO_2_ in films are not different for the same species in the different samples, the fraction of Sn atoms in each phase should follow the same trend, with annealing or with O_pp_ used. Indeed, the data recorded reveal that the fraction of Sn present as β-Sn is lower in the film deposited at higher O_pp_ (3.6%), than in those deposited at 3.0%. After annealing at 200 °C, the films crystallize leading to the formation of a strong α-SnO phase which also contributes to the oxidation of β-Sn. Under these conditions, the SnO_x_ with 1 < x < 2, is the dominant phase of the channel layer with a small contribution from metallic tin, explaining the p-type transport behaviour observed.

### 2.4. Electrical Data and Analysis

Hall Effect measurements were performed to identify the charge carrier and carrier mobility in the material. As we are in the presence of ambipolar material, as it is the case of SnO_x_ [64], electrons and holes will pile up at the same side of the sample and consequently the measured Hall voltage depends on the relative mobilities and concentrations of holes and electrons. Hall mobility for an ambipolar semiconductor is thus given by,

(1) $$\mu_{Hall}=\frac{n\cdot \mu_{n}^{2}-p\cdot \mu_{p}^{2}}{n\cdot \mu_{n}+p\cdot \mu_{p}}$$

where *n, p, µ_n_* and *µ_p_* represent electron density, hole density, electron mobility and hole mobility respectively. This leads to a reduction in the Hall mobility, compared to the mobilities of the charge carriers.

In the present study, the samples as deposited exhibit fluctuations in the sign and magnitude of the Hall coefficient, where the average mobilities of carriers were of about 10^−1^ cm^2^ V^−1^ s^−1^. After annealing up to 200 °C for 30 minutes, films prepared with 2.8% < O_pp_ <3.8 show a positive Hall coefficient. This suggests a considerably large density of holes compared to the density of electrons, resulting in a positive Hall voltage with typical Hall mobility of 2 cm^2^ V^−1^ s^−1^ associated to the materials’ evaluated. 

**Figure 2:**
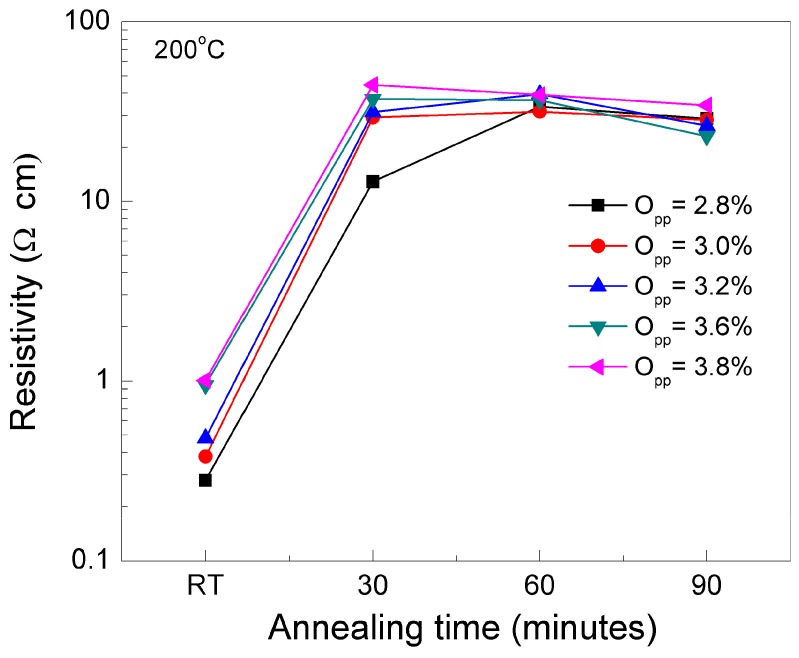
Resistivity of SnO_x_ films as a function of annealing time, for films processed at different O_pp,_ as depicted in the Figure.

Figure 2 shows the resistivity variation of SnO_x_ films for different O_pp_, as deposited and after annealing at 200 °C, for different annealing times.

The data show that the resistivity of the films processed tends to saturate around 30–40 Ω·cm, for annealing times above 30 mn. The lowest annealing time is for samples processed with O_pp_ above 3.20%, while the highest time (above 60 mn), for samples prepared with 2.80%< O_pp_ <3.20%. 

Annealing at temperatures above 200 °C cause again a decreasing tendency in material resistivity (not shown here) that can be associated with the phase transformation of the material from SnO (p-type) to SnO_2_ (n-type). These data are consistent with those depicted in Figure 1b.

### 2.5. Optical Data and Analysis

**Figure 3:**
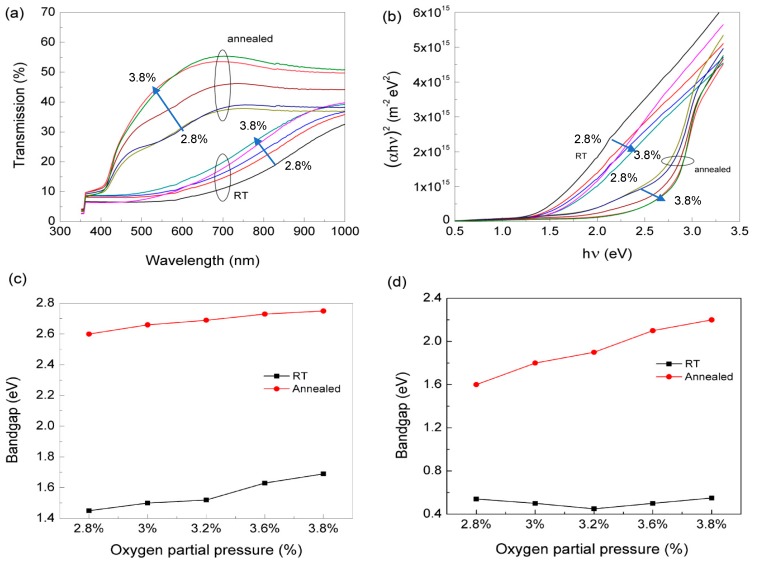
(**a**) Optical transmission of as prepared and annealed SnO_x_ films. (**b**) Plot for the estimation of the direct band gap estimation; Dependence of the estimated direct (**c**) and indirect (**d**) band gap on O_pp_ used, before and after annealing the films for 30 mn.

Figure 3a shows the optical transmittance data of the films processed for 2.80%< O_pp_ <3.20%, as deposited (RT) and after annealing at 200 °C, during 30 mn. The data depicted show that as O_pp_ increases the films become more transparent, as expected. As deposited, independent of O_pp_ used, the data depicted show, on average, transmittances below 20% in the visible region. These low values are attributed to the presence of large concentration of metallic tin in the films. By annealing the films, the optical transmittance in the visible region increases up to 55%, function of the O_pp_ used.

The optical band gap *E_g_* of the films were determined by the relationship:

(2) $$\alpha \cdot h\cdot \nu =const\cdot {\left(h\cdot \nu -E_{g}\right)}^{r}$$

where α is the absorption coefficient, *hν* denotes the photon energy and *r* a constant depending on the type of optical transition expected. The *E_g_* value is then obtained by linearly extrapolating the plot of ${\left(\alpha \cdot h\cdot \nu \right)}^{1/r}$ versus h⋅ν and finding the intersection with the abscissa. 

The optical data in Figure 3a were analysed with *r* = ½ (direct transition, for SnO, expected to be *E_g_* ~2.5 eV and for SnO_2_ *E_g_* ~3.6 eV, see Figure 3b) and *r* = 2 (Indirect transition, for SnO *E_g_* ~1 eV). For the different O_pp_ used, as deposited the direct band gap varies between 1.5 eV and 1.8 eV, while the estimated indirect band gap is kept around 0.6 eV. These values reflect the quasi metallic state of the films produced. After annealing, the films are better oxidized and the structure changes, as observed in Figure 1. Overall, we estimate a direct band gap with 2.6 eV <*E_g_* <2.75 eV, while the indirect band gap is 1.6 eV < *E_g_* < 2.2 eV (see Figure 3c,d). These values are close to the reported bandgap values for SnO films, showing for the range of O_pp_ used the SnO_2_ phase does not dominate the optical characteristics of the films produced. Small variations in these values could be understood on the basis of fractional variations of various phases (metallic tin, SnO and SnO_2_) in the films. 

## 3. Devices Results and Analysis

Taking into a count the set of results obtained during the evaluation of the films processed, we centred our attention in evaluating the devices performances by using tin oxide channel layers processed close to the extremes of the O_pp_ window in which a clear p-type behaviour was observed after annealing, respectively for O_pp_ = 3.0% (≈1.57 sccm) and O_pp_ = 3.6% (≈1.78 sccm). To reduce the channel conductance, thus allowing a better modulation of the same, we reduced the channel layer thickness to values around 12 nm. A batch of more than 40 devices were evaluated and the devices performances varied within a standard deviation of about ± 7% from the average.

### 3.1. Devices Structure, Geometry, Fabrication and Characterization Conditions

Bottom gate TFTs were fabricated on glass substrates coated with 150 nm thick layer of sputtered ITO and a 220 nm thick layer of aluminium-titanium oxide (ATO). SnO_x_ channel layer (width/length = 50 nm/50 nm and 12 nm thick) was deposited over this coating by r.f. magnetron sputtering at RT, using the same process conditions as reported before. Drain and source electrodes were based on Ni/Au (9 nm/60 nm) stack layers deposited by electron beam evaporation. After deposition, the devices were annealed in air up to 200 °C for 30 minutes.

**Figure 4:**
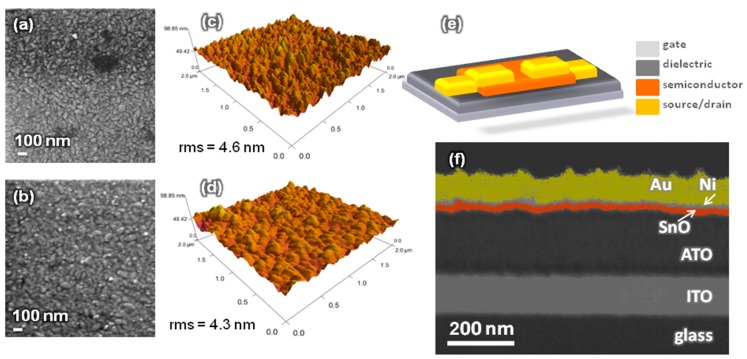
After annealing (60 mn) scanning electron microscope (SEM) images of SnO_x_ films processed at O_pp_ = 3.0% (**a**) O_pp_ = 3.6% (**b**) 3.6%, respectively. (**c**) and (**d**) show the corresponding AFM images. (**e**) Schematic illustration of SnO_x_ TFT structures fabricated. (**f**) Cross-sectional SEM image of the fabricated TFT.

Figure 4a–d show the SEM and AFM images of the surface morphology of the TFT channel layer 12 nm thick. The data depicted show that the shape of the microstructure of the grains obtained are similar, slightly increasing as O_pp_ increases, while the roughness decreases, respectively from 4.6 nm to 4.3 nm. 

Figure 4e is a schematic of the SnO_x_ TFT with a bottom gate configuration. Figure 4f shows the cross-section SEM image of the TFT prepared at 3.0% O_pp_, revealing a perfect step coverage of the deposited layer (channel and drain/source contacts), highly compact, uniform and homogeneous, without visible defects. 

TFT electrical characterization was performed with an Agilent 4155C semiconductor parameter analyzer (Santa Clara, CA, USA) and a Cascade Microtech M150 microprobe station (Livermore, CA, USA) inside a dark box at ambient atmosphere. By doing so, we avoid problems that may arise from persistent photoconductive effects [65].

### 3.2. Capacitance Measurements Data and Analysis

**Figure 5:**
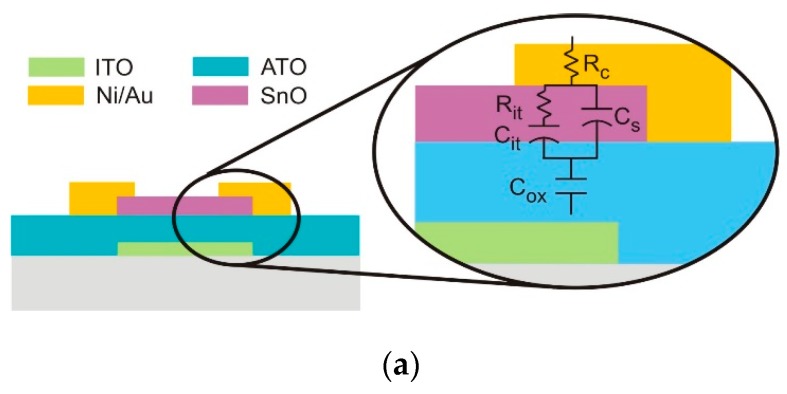

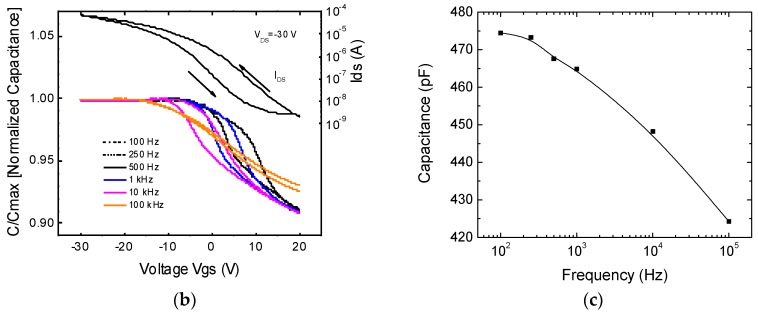
(**a**) Cross section sketch of the TFT, where t is also shown the equivalent electrical circuit based on RC resonators. (**b**) Normalized C-V plot taken for different frequencies (below) and the IV transfer characteristic of the TFT (above); (**c**) Dependence of the maximum capacitance (*C_max_*) achieved on the frequency used.

In order to better understand the role of the interfaces as well as of the dielectric on the TFT performances, CV measurements were directly performed on the TFTs by performing measurements between the gate electrode and drain and source short-circuited. The data were interpreted using an electrical model consisting of a contact resistance *R_C_* (the same value for drain and source regions) in series with a combination of two parallel RC resonators as shown in the sketch of Figure 5a. One of the resonators represents the semiconductor channel capacitance (*C_S_*) that varies dynamically, depending on the extension of the accumulation/depletion layer. The other component is the interface trap capacitance (*C_it_*) in series with the corresponding associated interface resistance *R_it_*, both depending on the interface defects given by:

(3) Cit=ρ(V)dVdx=εε0ρ(V)Q=q2Dit1+ω2τit2

where *D_it_* is the interface trap density, *τ_it_* = *R_it_* × *C_it_* is the trap response time. Finally, we have in series the insulator geometric capacitance (*C_ox_*). As the frequency tends to a steady state condition, we have almost *C_it_* in parallel with *C_s_* and the resulting capacitance in series with *C_ox_*. Therefore, for non-perfect semiconductors (basically, the amorphous ones), by using a frequency modulation less than the relaxation frequency of the semiconductor ($f_{r}=\frac{1}{2\pi R_{B}C_{s}}=\frac{1}{2\pi \rho_{B}\varepsilon_{S}\varepsilon_{0}}$, where *R_B_* is the channel bulk resistance), we expect that capacitance will be influenced by the number and nature of the interface defects, their length and depth extension which determines the way in which the structure responds to the electrical stimulus in the low frequency regime. On the other hand, at very high frequencies, *C_s_* dominates and *C_it_* *≅* *0,* being now relevant the role of *R_it_* in parallel with *C_ox_.* In this case the capacitance-voltage curves can be distorted due to charging effects, leading to a decrease or even to a not well-defined flat capacitance maximum (*C_max_*), as observed in the normalized C-V plots of Figure 5b for different frequencies and on the behaviour of *C_max_* with the frequency, depicted in Figure 5c. Figure 5b also shows the transfer characteristics of the TFT under analysis, where the hysteresis behaviour recorded is visible, which we associate with the role of interface defects. 

Figure 5c shows the dependence of the maximum capacitance recorded on the frequency. There, we notice that at frequencies below 100 Hz the capacitance tends to reach flat behaviour while the hysteresis voltage shift is enhanced, following a similar trend as that of the I-V TFT transfer characteristics. We associate this behaviour with interface localized states that respond at frequencies below *f_r_*. 

The analysis of the C-V plots shows that the minimum capacitance is not fully flat. This behaviour is attributed to the small thickness of the semiconductor (≤ 12 nm), which limits the extension of the depletion region. As the possible maximum width of depletion region is 12 nm (the semiconductor thickness), this means that the minimum capacitance of the system will not be much lower than the total capacitance. We also estimate the flat band voltage shift and the oxide charge density.

**Figure 6:**
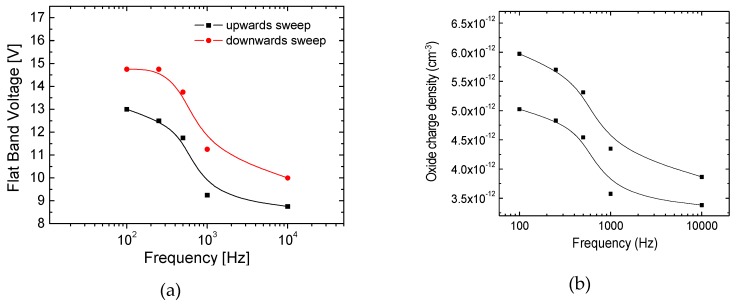
(**a**) Flat band Voltage dependence on the frequency; (**b**) Oxide charge density as a function of the frequency. The upper curves correspond to the upwards sweep while the lower curves to the downwards sweep (see Figure 5b).

Figure 6 shows the dependence of these parameters on the frequency, for the upward and downwards sweeps, in order to take into account the hysteresis observed. For the set of calculations performed, it was considered that the level of acceptor concentration (N_a_) was in the range of 10^17^ cm^−3^ with a good work function match between the gate electrode and the semiconductor.

Overall the data depicted show an enhancement on the flat band voltage and on the oxide charge density as the frequency decreases.

### 3.3. TFT Electrical Data and Analysis

**Figure 7:**
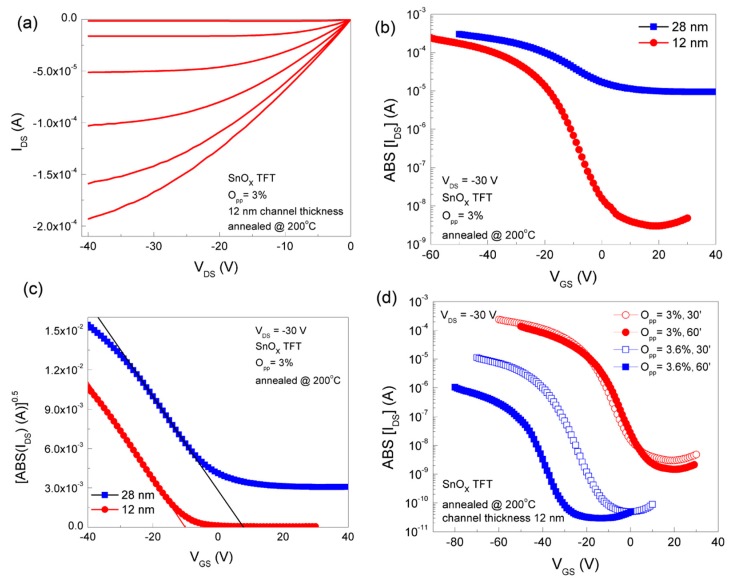
(**a**) Output characteristics of SnO_x_ p-channel TFT. The gate voltage is varied from 0 V to −50 V in −10 V steps. (**b**) Transfer characteristics of SnO_x_ p-channel TFT with different channel thickness. (**c**) Plot of square root of drain current, at V_DS_ = −30 V, for evaluating saturation mobility and threshold voltage. (**d**) Transfer characteristics of SnO_x_ p-channel TFT (channel thickness 12 nm) for different annealing time.

**Figure 8:**
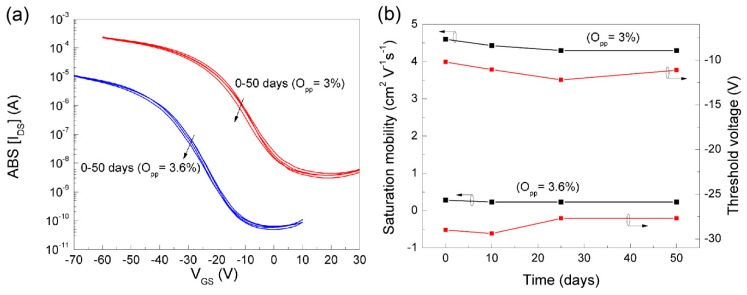
Transfer characteristics of SnO_x_ p-channel TFT measured at different days after device fabrication (**a**) and corresponding variations in saturation mobility and threshold voltage (**b**).

Figure 7 and Figure 8 show the TFT electrical characteristics of the two extreme cases evaluated, as processed, and after 50 days as a way to determine their stability (devices ageing effects).

Figure 7a shows the output characteristics (I_DS_-V_DS_) of such TFT produced at O_pp_ = 3.0%. The gate voltage was varied from 0 V to −50 V in −10 V steps. Very small I_DS_ at zero gate voltage indicates an almost closed channel. On increasing the gate voltage to higher negative values, holes accumulated at the channel-insulator interface forms a conduction path between source and drain. These TFTs exhibit hard saturation at large V_DS_, which is similar to pinch off in the usual field-effect transistors, revealing the high quality of the devices fabricated. 

Figure 7b shows the transfer characteristics of the same TFT, as above, but now using two different thicknesses for the channel layer, 12 nm and 28 nm respectively. The data depicted reveal that by decreasing the channel thickness the On/Off ratio improves from ~10^2^ to 7 × 10^4^, while the subthreshold swing voltage (the slop of the transfer curve when the current goes from the Off state to the On state) is reduced by more than a factor of 3. This allows us to select 12 nm as the best device thickness as stated before.

Figure 7c shows the plot of the square root of the absolute value of the drain current, at V_DS_ = −30 V as a function of V_GS_, for the same samples depicted in Figure 7b, for evaluating saturation mobility and threshold voltage, respectively. The data depicted show that irrespective of the channel layer thickness, the saturation mobility achieved is around 4.6 cm^2^ V^−1^ s^−1^, while the threshold voltage is shifted from 8.1 V to −10 V on reducing the channel thickness from 28 nm to 12 nm, respectively. Negative threshold voltage indicates the enhancement mode operation of the p-channel TFTs, as required to keep the channel closed when no voltage is applied to the gate.

Figure 7d shows the dependence of the absolute value of I_DS_ [ABS(I_DS_)] as a function of V_GS_ for devices fabricated using O_pp_ equal to 3% and 3.6%, respectively and heat treated at 200 °C during 30 mn and 60 mn, respectively. Table 4 shows the TFT parameters extracted from Figure 7d, where the dominant phase composition of the films processed is also shown, as revealed by the difractograms in Figure 1. The data show that the most stable devices with the best device performances are achieved when using an O_pp_ of about 3% and an annealing time of around 30 minutes, where the main dominant phase is just attributed to α-SnO. On the other hand, the films processed at O_pp_ ≥ 3.6% reveal a growing role of the β-SnO phase and so increase the ambipolar behaviour of the films processed.

Figure 8 shows the transfer curves of TFTs fabricated using O_pp_ = 3% and O_pp_ = 3.6%, measured along consecutive 50 days after their fabrication. The data show that that the TFTs are very stable in operation, without any significant variation on On/Off ratio, threshold voltage and mobility.

## 4. Discussion of the Results

The analysis of the structure, composition and morphology of the films processed, together with the evaluation of their electrical and optical performances, allow us to select the best process conditions to produce high stable and reliable p-type tin oxide films by RFMS at RT, surpassing the existing state of the art knowledge, as shown in Table 1. The data shown in Figure 1 reveal a structure in which the metallic tin dominates over the SnO phase, as also confirmed by Mössbauer spectroscopy data. This suggests that in the α-SnO phase, the Sn^2+^ oxide present is amorphous. After annealing in air, the metallic tin (β-Sn) is oxidized leading to the SnO_x_ (1 < x <2) phase formation, where now the Sn^2+^ oxide present is crystalline, with a strong α-SnO phase for which the minimum heat treatment time required is of about 30 min. To reach this goal O_pp_ of ≥2.8% is needed which corresponds with the use of an oxygen gas flow above 1.46 sccm. 

Below an oxygen gas flow of 1.46 sccm, films are metallic in nature and adhesion to the glass substrate was very poor. 

On the other hand, if O_pp_ > 3.8%, which corresponds with the use of an oxygen gas flow above 1.89 sccm, the films are highly resistive (>10^8^ Ω·cm), decreasing substantially after annealing, with SnO_2_ being the dominant phase. In these conditions, films exhibit an n-type conduction behaviour. This clearly favours the ambipolar behaviour, as broadly observed [38,42,51,53].

Since we used a metallic tin target for sputtering, very low O_pp_ was not sufficient to oxidize the film. The experimental data show that a stable SnO phase is only obtained if used during the deposition process of an oxygen gas flow in the range of 1.46–1.89 sccm (2.8% < O_pp_ <3.8%.), after heat treatment at 200 °C for 30 minutes. This corresponds to producing films with a resistivity in the range of 30–40 Ω.cm (see Figure 2). On the other hand, the use of high oxygen gas flow (more than 1.89 sccm) favours SnO_2_ formation. That is, we can use the same material and, by proper control of the process parameters such as O_pp_ and gas flow, turn it into an n-type or p-type semiconductor [51,53].

The optical data shows that the transparency in the visible range for films as deposited is below 20%, increasing as O_pp_ increases. Moreover, the heat treatment enhances the optical transparency by about 3 times, proving that under these conditions the oxidation phase dominates over the metallic one (see Figure 3). The estimated bandgap value for the films deposited using oxygen flow in the range of 1.46–1.89 sccm was around 2.6 eV (see Figure 3). This agrees with the reported direct bandgap value of 2.7 eV, associated with the SnO dominant phase. 

The study described above allowed us to select the best process conditions to grow p-type tin oxide films as being those for which O_pp_ = 3%. Moreover, allow us also to observe that the thickness of the films plays a relevant role in determining the electrical modulating behaviour of the channel, as also observed in other works [48]. This led us to select the proper thickness of the channel layer as being 12 nm, as shown in Figure 4. 

The analysis of the capacitance measurements realized on the TFT (pad contact area of about 100 μm × 100 μm) shows that the capacitance reaches a flat maximum for frequencies below 100 Hz, which corresponds to a value of about 23.8 nF/μm^2^. This allowed us to infer the saturation mobility [15]. Here, the reduced channel thickness (≤ 12 nm), limits the extension in which the channel is depleted and also its modulation extension. The hysteresis observed is attributed to the role of interface localized states in responding to electrical stimulus below the relaxation frequency *f_r_*. Moreover, from these measurements we could estimate the flat band voltage shift and the oxide charge density (see Figure 6) as being in the range from 13–15 V and 5 × 10^12^–6 × 10^12^ C/cm^3^, for the low frequency regime, shifting towards 9–10 V and 3.5 × 10^12^–4 × 10^12^ C/cm^3^, respectively for the high frequency regime, where shallow states do not respond to the electrical stimulus [15].

The analysis of the electrical characteristics of the TFT depicted in Figure 7 and Figure 8 show that p-channel oxide TFTs manufactured at RT, followed by intentional heat treatment at 200 °C, using a narrow process window associated with the oxygen gas flow used are highly reproducible, reliable and stable. In addition, they all work in the enhancement mode, exhibiting very good electronic performance, translated by high mobility and On/Off ratio, together with a relative low threshold voltage. The annealing temperature used is suitable for fabricating these devices on plastic or even on paper substrates [7], which cannot be the case when using heat treatment much above 200 °C [29,30,31,32]. The data depicted clearly show that the films developed throughout this study are among the best ever produced, as can be seen in Table 1 and Table 2, irrespective of the process technique used. Moreover, the very high On current of these devices makes them ideal for fabricating active matrix OLED driving circuits, where a TFT must supply sufficient hole current to the anode of the OLED. Use of an n-channel TFT results in a voltage drop over the OLED, which affects the drain current of the TFT. However, it does not affect the drain current of a p-channel TFT in the saturation mode. 

## 5. Conclusions

In summary, we have fabricated highly reproducible high-performance p-channel oxide TFTs on glass substrates using an SnO_x_ channel layer, for which the proper process conditions were selected. The SnO phase was identified and quantified by two independent techniques, XRD and Mossbauer spectroscopy, corroborating the p-type oxide semiconductor behaviour obtained, for films grown at low O_pp_ (around 3%), where the dominant phase is due to α-SnO. The TFTs fabricated show typical saturation mobility of 4.6 cm^2^ V^−1^ s^−1^ and On-Off ratio > 7 × 10^4^, with threshold voltages of about −10 V, indicating that the devices work in the enhancement mode. This is one of the best stable electrical performances achieved so far for p-type oxide TFTs processed at RT and heat treated at temperatures around 200 °C, as the survey conducted concerning the present state of the art in producing p-type TFT based on tin oxide shows (see Table 1 and Table 2). This will enable the fabrication of fully transparent CMOS either on rigid or flexible substrates, associated with all the advantages offered by transparent/oxide electronics.

## Figures and Tables

**Table 1:** Recent developments concerning the performances of p-type SnO TFTs processed by Radio Frequency Magnetron Sputtering (RFMS), using different type of device configurations, O_pp_, dielectrics and process temperatures.

Method	Device Structure	Process Temp. (°C)	O_pp_ (%)	Dielectric	μ_h,FE_ (cm^2^ V^−1^ s^−1^)	I_on_/I_off_	Year	Ref.
RFMS	SBG	RT *	11.5	ATO	1.2	10^3^	2010	[28]
RFMS	SBG	300	1	SiN_x_	0.24	10^2^	2010	[32]
RFMS	SBG	150	n.r.	Paper	1.3	10^2^	2011	[33]
RFMS	SBG	250	n.r.	SiO_2_	1.8	10^3^	2013	[34]
RFMS	SBG	225	4.3	HfO_2_	0.33	10^3^	2014	[35]
RFMS	SBG	230	n.r.	SiO_2_	0.59	3 × 10^3^	2014	[36]
RFMS	CTG	200	9	P(VDF-TrFE)	3.3	3 × 10^2^	2014	[37]
RFMS	SBG	200	11.8	SiO_2_	1.36	2 × 10^3^	2014	[38]
RFMS	STG	200	9	P(VDF-TrFE)	2.7	2 × 10^2^	2014	[39]
RFMS	SBG	200	n.r.	SiO_2_	0.61	6.2 × 10^5^	2015	[40]
RFMS	SBG	250	n.r.	SiO_2_	1.8	10^5^	2015	[41]
RFMS	SBG	200	7.5	SiO_2_	4.13	6 × 10^2^	2015	[42]
RFMS	SBG	250	-	SiO_2_	1.16	2.3 × 10^2^	2015	[43]
RFMS	SBG	300	n.r.	SiO_2_	3.33	10^4^	2016	[44]
RFMS	SBG	200	n.r.	SiO_2_	0.63	5.2 × 10^6^	2017	[45]
RFMS	SBG	225	n.r.	Al_2_O_3_	0.7	2.6 × 10^4^	2018	[46]
RFMS	SBG	120	9	HfO_2_	5.53	2.7 × 10^3^	2018	[47]
RFMS	SBG	225	3.1	SiO_2_	1.41	1.5 × 10^3^	2018	[48]
RFMS	SBG	225	3.1	SiO_2_	0.87	1.88 × 10^4^	2019	[49]
RFMS	SBG	RT *	3.0	ATO	4.6	7 × 10^4^	2019	This work

* Post deposition annealed at 200 °C; n.r.: not reported; RT = Room Temperature; ATO = Aluminum Titanium Oxide.

**Table 2:** Recent developments concerning the performances of p-type SnO TFTs processed by other physical and chemical process techniques, for different device configurations, O_pp_, dielectrics and process temperatures.

Method	Device Structure	Process Temp. (°C)	Oxygen Partial Pressure (%)	Dielectric	μ_h,FE_ (cm^2^ V^−1^ s^−1^)	I_on_/I_off_	Year	Ref.
PLD	STG	575	4 × 10^−2^ Pa	Al_2_O_3_	1.3	10^2^	2008	[50]
PLD	STG	250	1 × 10^−2^ Pa	SiO_2_	0.81	~10^2^	2011	[51]
PLD	SBG	300	1 × 10^−2^ Pa	SiO_2_	2.18	--	2014	[52]
EBE	SBG	400	n.r.	SiO_2_	0.32	5 × 10^2^	2013	[53]
TE	SBG	250	n.r.	Al_2_O_3_	1.4	5 × 10^4^	2018	[54]
DCMS	SBG	180	9	HfO_2_	6.75	~10^3^	2013	[29]
DCMS	DG	300	3.07 × 10^−2^ Pa	SiO_2_	6.54	10^5^	2015	[30]
DCMS	SBG	200	n.r.	HfO_2_	5.56	4.8 × 10^4^	2016	[55]
DCMS	SBG	200	n.r.	HfO_2_	7.6	3 × 10^4^	2018	[31]
PVD	SBG	200	n.r.	HfO_2_	2.13	9.6 × 10^6^	2017	[56]
ALD	SBG	250	n.r.	Al_2_O_3_	1	2 × 10^6^	2017	[57]
SC	SBG	450	-	SiO_2_	0.13	85	2012	[58]

* Post deposition annealed at 200 °C; n.r.: not reported.

**Table 3:** Extrapolated parameters from the transmission ^119^Sn Mössbauer spectra of bulk samples and CEMS spectra of films taken at RT.

Sample	IS (mm/s)	QS (mm/s)	Γ (mm/s)	Sn Phase	I (%)
SnO_2_ bulk	0.01	0.56	1.34	-	100
α-SnO bulk	2.67 −0.03	1.34 0.58	0.98 0.77	α-SnO SnO_2_	95 5
-Sn metal	2.56	-	1.03	-	100
Film O_pp_ = 3.0%, RT	2.76 0.11 2.56	1.87 0.52 −	1.04 0.84 1.3	SnO SnO_2_ -Sn	65 15 20
Film O_pp_ = 3.0%, 200 °C, 30 min	2.7 0.08 2.56	1.38 0.59 −	0.79 0.7 1.35	SnO SnO_2_ -Sn	81 8 11
Film O_pp_ = 3.6%, RT	2.73 0.11 2.56	1.82 0.53 −	1.02 0.81 1.3	SnO SnO_2_ -Sn	78 15 7
Film O_pp_ = 3.6%, 200 °C, 30 min	2.7 0.06	1.34 0.48	0.82 0.85	SnO SnO_2_	88 12

IS (mm/s) isomer shift relative to metallic BaSnO_3_ at 295 K; QS (mm/s) quadrupole splitting; Γ (mm/s) line-width; I relative area. Estimated errors ≤0.02 mm/s for IS, QS, Γ and <2% for I.

**Table 4:** Electrical properties of SnO_x_ p-channel TFTs for different annealing time (channel thickness 12 nm). After annealing all structures are polycrystalline.

Annealing Conditions	Oxygen Partial Pressure (%)	Field Effect Mobility (cm^2^ V^−1^ s^−1^)	Saturation Mobility (cm^2^ V^−1^ s^−1^)	Threshold Voltage (V)	On-Off Ratio	Dominant Composition Phase
200 °C, 30 min	3.0	3.3	4.6	−10	7 × 10^4^	α-SnO
200 °C, 60 min	3.0	2.2	2.6	−7.2	9 × 10^4^	α-SnO
200 °C, 30 min	3.6	0.16	0.28	−29	2 × 10^5^	α-SnO + β-SnO
200 °C, 60 min	3.6	0.02	0.03	−38.6	3 × 10^4^	α-SnO + β-SnO
